# Lysyl oxidase inhibitors attenuate cyclosporin A-induced nephropathy in mouse

**DOI:** 10.1038/s41598-021-91772-5

**Published:** 2021-06-14

**Authors:** Long T. Nguyen, Sonia Saad, Ying Shi, Rosy Wang, Angela S. Y. Chou, Anthony Gill, Yimin Yao, Wolfgang Jarolimek, Carol A. Pollock

**Affiliations:** 1 Renal Medicine, Kolling Institute, Royal North Shore Hospital, University of Sydney, Sydney, NSW Australia; 2 Cancer Diagnosis and Pathology Research Group, Kolling Institute, Royal North Shore Hospital, University of Sydney, Sydney, NSW Australia; 3 Drug Discovery Department, Pharmaxis Ltd., Sydney, NSW Australia

**Keywords:** Chronic kidney disease, Renal fibrosis, Drug development

## Abstract

Calcineurin inhibitors, such as Cyclosporin (CsA), are the mainstay of anti-rejection therapy in solid organ transplants but can paradoxically induce progressive nephropathy characterised by renal dysfunction and interstitial fibrosis. Lysyl oxidases (LOXs), a group of enzymes that catalyse extracellular matrix (ECM) crosslinking, were shown to implicate in tissue scarring. It is hypothesized that inhibition of these enzymes may render therapeutic effects against CsA-induced nephropathy. In this study, 6-to-8 weeks old C57BL/6 J mice were administered saline or CsA (30 mg/kg/day s.c) for 16 weeks. At 8 weeks, CsA-treated animals were divided into 5 groups respectively treated with: (1) vehicle, (2) PXS-5505 (Pan-LOX inhibitor), (3) PXS-5382 (LOX-like 2 inhibitor), (4) PXS-5505 for 4 weeks then PXS-5382 for 4 weeks (sequential therapy), and (5) Telmisartan (standard therapy). Our results indicate that CsA administration significantly increased the levels of blood urea nitrogen, glomerular and tubular injury, tubulointerstitial fibrosis, inflammation and oxidative stress in mouse kidney. These changes were associated with upregulated mRNA expression of LOX and LOXL2. Administration of Pan-LOX or LOXL2 inhibitors or the sequential therapy suppressed the expression of ECM proteins (α-SMA, FN and COL1A), matrix metalloproteases (MMP)2 and 9, inflammatory markers (TNFα and MCP-1) and TGF-β1-Smad3 signalling. Among all regimens including telmisartan, only Pan-LOX inhibitor PXS-5505 was able to attenuate uraemia. Collectively, our study suggests that Pan-LOX and LOXL2 inhibition can attenuate progressive nephropathy due to CsA administration.

## Introduction

Calcineurin inhibitors (cyclosporin and tacrolimus) are the mainstay of anti-rejection therapy in renal transplantation but paradoxically are the main cause of chronic allograft nephropathy. Toxicity has been documented as early as within the first 3 months after transplantation^1,2^. Kidney fibrosis and renal functional decline are observed in 50% of patients one year post transplantation and become almost universal at 10 years^2,3^. Cyclosporin A (CsA) in particular is also implicated in the development of nephrotoxicity in patients with heart and liver transplant as well as in patients with autoimmune disease without a previous history of nephropathy^4–6^. Nephrotoxic side effects and the inherent requirement for monitoring of blood pressure levels have limited the clinical utility of calcineurin inhibitors and led to the development of alternative anti-rejection strategies, including CsA-dose reduction combined with administration of anti-inflammation (e.g. prednisone) and anti-proliferative agents (e.g. mycophenolate mofetil). However, these treatments can result in insufficient immunosuppression, leading to limited short- and long-term benefits to graft survival and nephrotoxicity^7,8^.

Most data on calcineurin inhibitors-induced nephrotoxicity pertain to cyclosporin since it has been used for a much longer time. While the mechanisms underlying CsA nephrotoxicity are still not fully understood, a typical histopathological hallmark of chronic nephropathy is tubulointerstitial fibrosis, which can be fundamentally described as the deposition and cross-linking of extracellular matrix (ECM) proteins such as fibronectin and collagens. CsA has been shown to upregulate interstitial collagens expression by multiple discreet kidney cells isolated from rats and humans, especially fibroblasts^9^, as well as to induce epithelial–mesenchymal transition (EMT) in human renal proximal tubular epithelial cells^10^. In animals, CsA administration induces interstitial deposition of collagen type III and fibrosis via stimulation of the Transforming Growth Factor beta (TGF-β)-signalling pathway^11^, while inhibiting ECM degradation via modulation of matrix metallopeptidase 9^12^, thus leading to an imbalance in ECM turn over.

Lysyl oxidases (LOXs) and lysyl oxidase‐like 1‐4 (LOXL 1‐4) are a group of enzymes that catalyse cross-linking of collagens, thereby rendering these ECM proteins insoluble and unable to be degraded^13,14^. In particular, the expression of LOX and LOXL2 have been shown to positively correlate with fibrosis in the multiple organs including kidney^15,16^. Indeed, recent studies have shown that LOXL2 inhibition can lead to marked reductions in activated fibroblasts^17,18^, reduced collagen content and crosslink formation and reversal of fibrosis in these tissues^19–21^. It is unclear whether LOX/LOXL2 inhibition is also beneficial in attenuating the development of CsA nephrotoxicity. As such, this project aimed to examine the therapeutic effectiveness of two inhibitors PXS-5505 and PXS-5382, which respectively target all members of the LOX family (pan-LOX) or only LOXL2, in a mouse model of CsA nephropathy. In addition to the use of the two drugs independently, we also experimented a sequential therapy starting with pan-LOX followed by LOXL2 inhibitor. As too much suppression of LOXs can affect healthy ECM turnover, the idea of the sequential approach is to inhibit all LOX isoforms at the initial phase of fibrosis formation, then switch to a more specific, moderate inhibitor to maintain a certain level of ECM that is necessary for systemic scarring/healing processes^22^.

## Methods

### Animals

The study was approved by the Animal Care and Ethics Committee of Northern Sydney Local Health District (RESP/18/318). All methods were performed in accordance with the relevant guidelines and regulations in the Australian Code of Practice for the Care and Use of Animals for Scientific Purposes, as well as in compliance with ARRIVE guidelines. Six-to-eight weeks old C57BL/6 mice were treated with daily subcutaneous injections of CsA (30 mg/kg/day) for 16 weeks with free access to standard diet and tap water^11^. At 8 weeks of CsA administration, the mice were administrated PXS-5505 (20 mg/kg/day) or PXS-5382 (30 mg/kg/day) for 8 weeks by oral gavage under short inhalational anaesthesia. Alternatively, a sequential treatment of PXS-5505 (20 mg/kg/day for 4 weeks) then PXS-5382 (30 mg/kg/day for 4 weeks) was undertaken. Blockade of the renin angiotensin aldosterone system (RAAS) has been the most validated intervention to reduce renal fibrosis^23^. Hence telmisartan (3 mg/kg/day in drinking water) was used as a best practice comparator to the LOX inhibitor strategies. Mice were sacrificed by cardiac puncture during deep anaesthesia. Blood was collected in EDTA-tubes (Becton Dickinson, NJ, USA) and centrifuged at 1,500 × g for 10 min for plasma collection. Kidney and other tissues were dissected, snap-frozen and stored in -80 °C; or they were fixed in neutral-buffered formalin (10%) for histological assessment.

### Biomarker assays of kidney function and damage

The blood level of urea nitrogen (BUN) was measured by BUN Colorimetric Detection Kit (Thermo Fisher Scientific, MA USA) and the plasma kidney injury marker (KIM-1) was measured by ELISA (Abcam, Cambridge, UK) as per the manufacturers’ instructions. 24-h urine was collected from metabolic cages prior to sacrifice and examined for creatinine (Cayman, MI, USA) and albumin (Abcam, Cambridge, UK) according to the manufacturers’ instructions, from which the urinary albumin to creatinine ratio (UACR) was calculated.

### RNA extraction and quantitative RT-PCR

Total RNA of renal tissues was isolated using RNeasy Plus Mini Kit (Qiagen Pty Ltd, CA, USA) according to the manufacturer’s instructions. The purified total RNA was used as a template to generate cDNA using the QuantiNova Reverse Transcription Kit and the amplicons of the target genes were amplified using QuantiNova SYBR Green PCR Kit (Qiagen Pty Ltd, CA, USA). The PCR reaction was performed by ABI Prism 7900 HT Sequence Detection System (Applied Biosystems, CA, USA). Primer sequences were summarized in Supplementary Table S1. Gene expression was standardized to β-actin mRNA and log-transformed.

### Protein extraction and immunoblotting

The tissues were homogenized and extracted in RIPA Lysis and Extraction Buffer using TissueRuptor (Qiagen, Hilden, Germany). Halt Protease and Phosphatase Inhibitor Cocktail (Thermo Scientific, MA, USA) was added to the extraction buffer. The protein concentration was measured using Pierce BCA Protein Assay Kit (Thermo Fisher Scientific, MA, USA) according to the manufacturer’s instructions. Protein aliquots containing the same amount of protein were prepared and frozen at -80 °C for later analysis. The protein samples were run on NuPAGE Tris–Acetate 3–8% gel (Thermo Fisher Scientific) for detection of Fibronectin, Collagen I and MCP-1 under the native conditions; while Bolt Bis–Tris 4–12% gel system (Thermo Fisher Scientific) was used for detection of other proteins under denatured conditions. Gels were electroblotted onto the Hybond nitrocellulose membrane (Amersham Pharmacia Biotech, Amersham, UK), and target proteins were captured by the corresponding primary-secondary antibody pairs. Primary antibodies, which have been validated in our previous studies^24,25^, are listed in Supplementary Table S2. β-actin was used for loading control. Luminata Western HRP Substrate (Millipore, MA, USA) was used for signal development and ImageQuant LAS 4000 (Fujifilm, Tokyo, Japan) was used for imaging. ImageJ (National Institutes of Health, USA) was used for densitometric quantitation of the protein expression.

### DNA extraction and 8-OHdG quantitation

DNA was extracted using Isolate II Genomic DNA Kit (Bioline, London, UK), which was then denatured by heating at 95 °C for 5 min then rapidly cooled to 4 °C. Nuclease P1 enzyme (New England Biolabs, MA, USA) was added to the sample and incubated at 37 °C for 30 min then 75 °C for 10 min to break the DNA into single nucleotides, which were then dephosphorylated by incubation with FastAP Thermosensitive Alkaline Phosphatase (Invitrogen, MA, USA) at 37 °C for 10 min. The enzyme was deactivated by heating at 75 °C for 5 min. DNA oxidative damage was assessed by measuring the level of 8-hydroxy-2'-deoxyguanosine (8-OHdG) using 8-hydroxy 2 deoxyguanosine ELISA Kit (Abcam, Cambridge, UK) following the manufacturer’s instructions.

### Histology

Paraffin embedded kidneys Sects. (4 μm) were stained with Pico-Sirius Red (PSR), Masson’s Trichrome (MT) and PAS stains respectively. For each sample, six random non-overlapping fields were captured at 200 × magnification using a bright-field microscope (Leica Microsystems, Wetzlar, Germany). The pathological scoring was performed by two independent examiners. For PSR, the area of staining was estimated by Image J software. The final PSR index (COL1 and COL3 abundance) was calculated by multiplying the intensity with the area of staining as per previously described^26^. Tubular dilation, interstitial inflammation and fibrosis were assessed by MT staining. Each characteristic of tubular injury was graded on a scale of zero to four based on the percentage of affected area as previously reported^21^: Grade 0 = no damage/staining; grade 1 =  < 25% area, grade 2 = 25–50% area; grade 3 = 50–75% area and grade 4 =  > 75% area. Ten non-overlapping fields were assessed, and the scores were averaged. PAS staining was used to score glomerulosclerosis (grade zero to four) based on the level of mesangial expansion and basement membrane thickening as per previously described^27^. A total of twenty glomeruli were randomly selected and the whole kidney average glomerulosclerosis index was obtained by averaging scores from all counted glomeruli in one section.

### Immunohistochemistry

Immunohistochemistry (IHC) staining was performed as previously described^28^. Briefly, tissues were fixed in 10% formalin for 36-h and embedded in paraffin or frozen-embedded in OCT solution (Tissue-Tek). Paraffin sections were prepared at a 4-μm thickness and mounted on microscope slides (Trajan Scientific and Medical, VIC, Australia). Antigen retrieval was performed at 99 °C for 20 min in 0.01 M citric buffer, pH 6.0. Endogenous peroxidase was deactivated with 3% H_2_O_2_ (Sigma-Aldrich, Dublin, Ireland). The slides were then blocked by Protein Block Serum-Free (Dako, Glostrup, Denmark), and incubated with α-SMA (A2547, 1:20,000, Sigma-Aldrich, MIS, USA,) at 4 °C overnight. After washing with TBST for 5 min, the slides were incubated with biotinylated secondary anti-rabbit IgG antibodies (Dako) for 30 min. The slides were washed again with TBST before staining with HRP-conjugated streptavidin (Dako) for 10 min. Using a light microscope (Leica DM750 photomicroscope with ICC50W digital camera), six consecutive non-overlapping fields from each kidney section were photographed and the percentage of staining area was quantitated using the Image J software (National Institutes of Health, USA).

### Statistical analysis

Data are expressed as mean ± SEM except for qRT-PCR results, which are presented as box plots with whiskers ranging from min to max. The data were analysed by Graphpad Prism 8.0 software. To validate the CsA nephropathy model (Ctrl vs CsA), unpaired t-test was used. To test the hypothesis that pan-LOX and LOXL2 inhibitors reduce CsA nephropathy (CsA vs CsA + treatments), we used one-way ANOVA followed by Benjamini post-hoc test, which controls for False Discovery Rate. *P* < 0.05 was considered significant.

## Results

### Pan-LOX but not LOXL2 inhibition reduced CsA-induced uraemia

**Figure 1:**
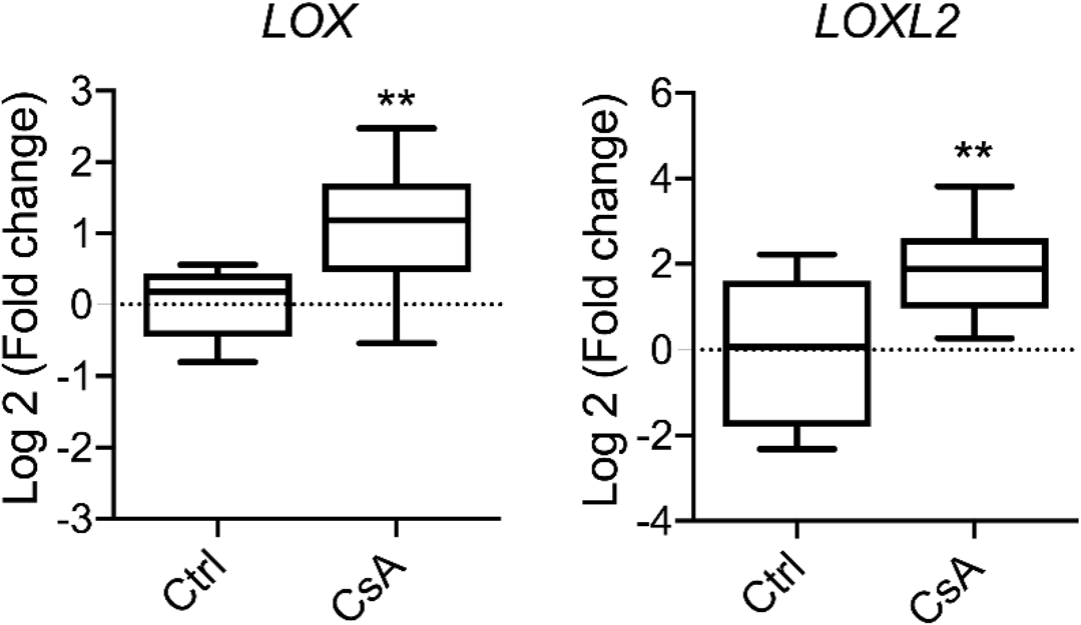
Renal mRNA expression of *LOX* and *LOXL2* is upregulated by cyclosporine A administration. n = 7–10. Data are presented as box plots with whiskers ranging from min to max. Unpaired t-test: **P < 0.01.

As expected, chronic administration of CsA resulted in upregulated mRNA expression of both *LOX* and *LOXL2* in the kidney (*P* < 0.01, Fig. 1). BUN was significantly increased following CsA administration reflecting kidney dysfunction (*P* < 0.001, Table 1). Neither the plasma level of kidney injury marker (KIM)-1 nor the urinary albumin/creatinine ratio (UACR) was elevated in CsA treated mice compared to other groups. There were also no differences in the animals’ body weight and kidney weight between CsA-treated animals compared to the control and treatment groups (Table 1). The elevation of BUN was significantly suppressed only by Pan-LOX inhibitor PXS-5505 (*P* < 0.05), while the other treatments showed no effect. Telmisartan showed a trend to exacerbate BUN. No significant effects were found by any of the treatments with respect to UACR or blood KIM-1 levels.

**Table 1:** Body weight, kidney weight, UACR, urinary KIM-1 and BUN levels.

	Ctrl	CsA	CsA + 5505	CsA + 5382	CsA + 5505/5382	CsA + tel
**BUN (mg/dL)**						
Mean	8.768	17.80***	12.02#	15.99	16.61	19.87
SE	0.740	1.895	1.216	1.130	1.255	1.637
**Plasma KIM-1 (pg/ml)**						
Mean	44.12	43.03	28.44	44.62	47.69	30.71
SE	0.1455	0.277	0.258	0.504	0.284	0.332
**UACR (mg/g)**						
Mean	12.82	10.37	14.10	13.03	12.33	11.99
SE	2.219	0.887	1.941	1.098	1.165	2.296
**Body weight (g)**						
Mean	28.11	26.42	25.73	25.75	24.49	26.43
SE	0.36	0.50	0.84	0.93	0.88	0.59
**Kidney weight (%BW)**						
Mean	1.416	1.363	1.383	1.288	1.494	1.36
SE	0.032	0.025	0.095	0.054	0.056	0.038

*UACR* urinary albumin creatinine ratio, *KIM-1* kidney injury marker 1, *BUN* blood urea nitrogen, *CsA* cyclosporine, *Tel* telmisartan. Vs Ctrl: ***P < 0.001; vs CsA: #*P* < 0.05. n = 7–10.

### The effects of Pan-LOX and LOXL2 inhibitors on CsA-induced renal histopathological changes

**Figure 2:**
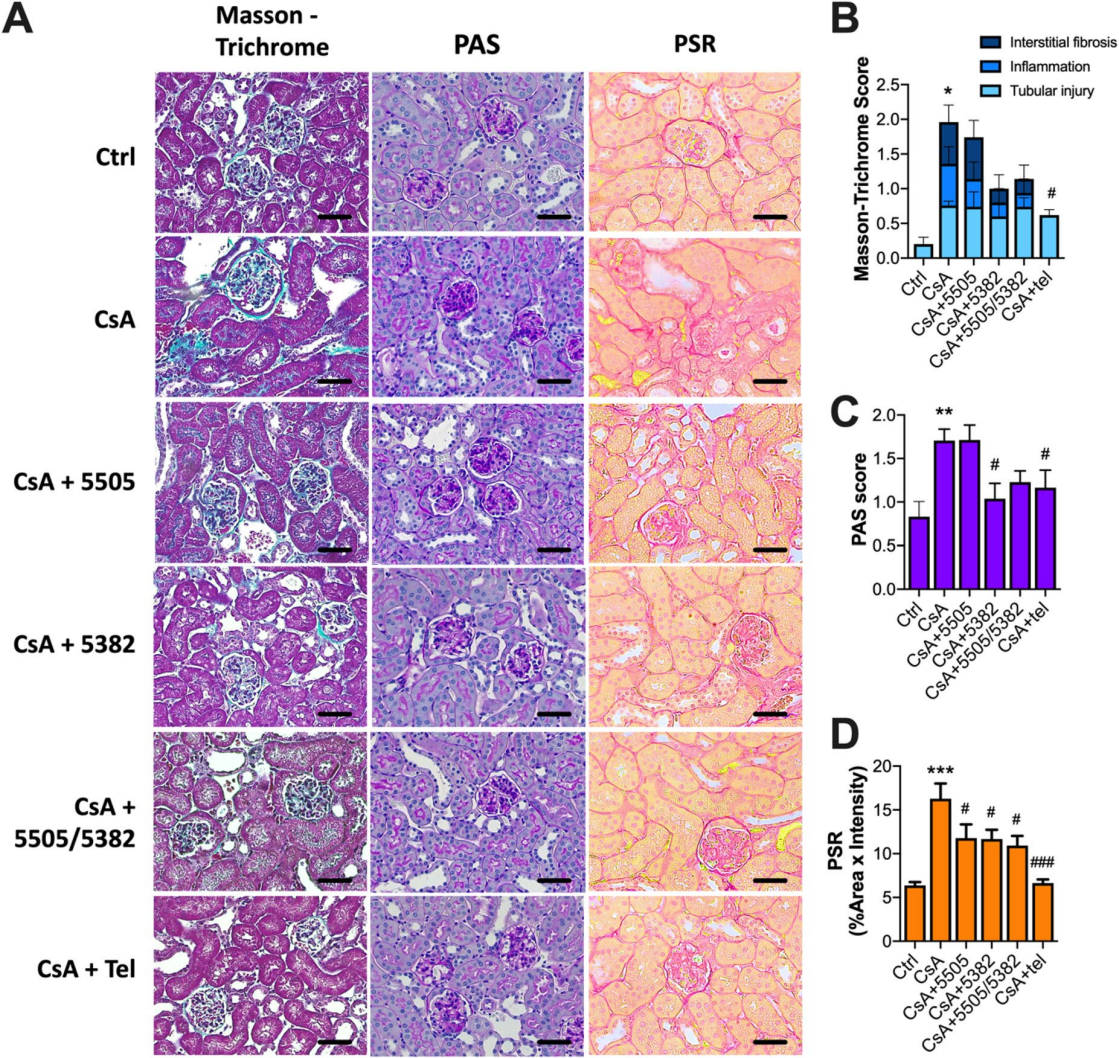
Histopathological assessment of kidney in mice treated with pan-LOX and LOXL2 inhibitors. (**A**) Representative images of histological staining; (**B**) Masson-Trichrome score reflecting tubular dilatation, inflammation and fibrosis (n = 5); (**C**) PAS score reflecting glomerulosclerosis (n = 7); (**D**) Pico-Sirius Red (PSR) staining (n = 7–10) . Data are expressed as Mean ± SEM. Vs Ctrl: *P < 0.05, **P < 0.01, ***P < 0.001; vs CsA: #*P* < 0.05, ###*P* < 0.001. Scale bar = 200 μm.

In parallel with uraemia, CsA administration also induced significant histopathological changes in the kidney. As can be seen from Fig. 2A,B, tubular injury, interstitial inflammation and fibrosis scores were elevated in the kidney of CsA-treated animals (all *P* < 0.05). PAS scores were also significantly higher in the CsA group (*P* < 0.01, Fig. 2C), indicating glomerulosclerosis. In addition, the level of PSR staining, which reflects the deposition of collagen type I (COL1) and type III (COL3) in the renal ECM, was also significantly increased following CsA exposure (*P* < 0.001, Fig. 2D).

Pan-LOX inhibitor PXS-5505 had no effect on tubular injuries, while LOXL2 inhibitor PXS-5382 and the sequential treatment (PXS-5505/5382) showed trends toward reduction in renal inflammation and tubulointerstitial fibrosis. Telmisartan also significantly abolished renal inflammation and fibrosis (*P* < 0.05, Fig. 2B). Similar to Masson-Trichrome result, no effect on PAS score was found in animals treated with PXS-5505 (Fig. 2C), suggesting no improvements with regard to glomerular damage. On the other hand, this was significantly reduced by PXS-5382 monotherapy (*P* < 0.05) and telmisartan (*P* < 0.05). The sequential therapy also reversed glomerulosclerosis to control level; however, this effect was not significant. As reflected by PSR staining, COL1/COL3 protein expression was significantly suppressed by all regimens (*P* < 0.05, Fig. 2D) and the change appeared to be most significant in those treated with Telmisartan.

### Pan-LOX and LOXL2 inhibitors suppressed myofibroblast activation and the deposition of extracellular matrix proteins

**Figure 3:**
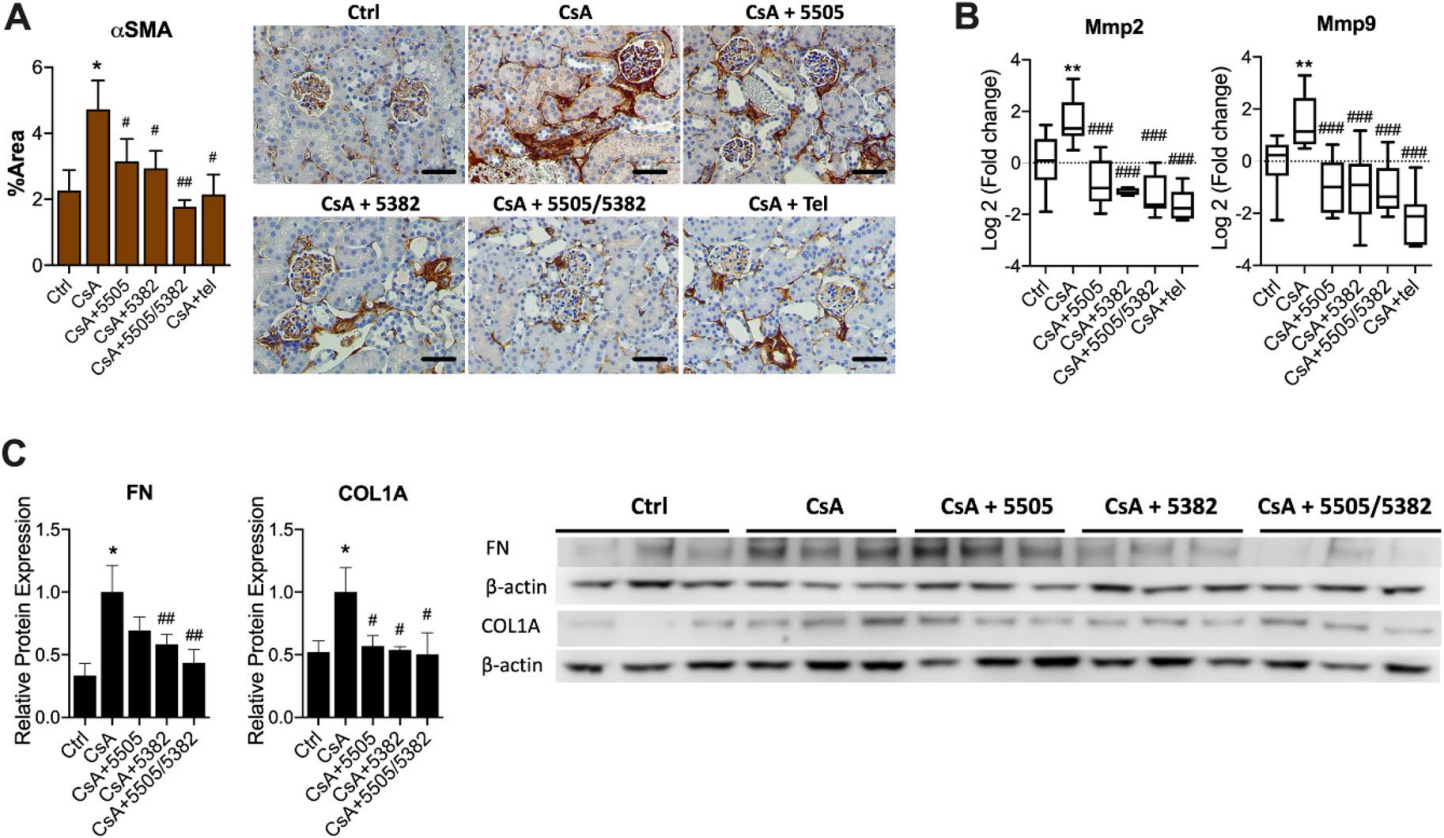
LOX and LOXL2 inhibitors suppressed fibroblast activation and the deposition of extracellular matrix proteins. (**A**) Immunohistochemistry of α-smooth muscle actin (n = 7). (**B**) mRNA expression of matrix metalloproteinase (MMP)2 and 9 (n = 7–10). C) Representative immunoblots and protein expression of fibronectin and collagen type 1A (n = 6). Data are expressed as Mean ± SEM for A and C, or as box plots with whiskers ranging from min to max for B. Vs Ctrl: *P < 0.05, **P < 0.01; Vs CsA: #*P* < 0.05, ##*P* < 0.05, ###*P* < 0.001. Scale bar = 200 μm.

Myofibroblasts are considered to be the major source of extracellular matrix proteins, which is the hallmark of renal fibrosis^29^. In this study, the expression of alpha-smooth-muscle actin (α-SMA), a specific marker of myofibroblast, was significantly increased in the kidney of animals treated with CsA (*P* < 0.01, Fig. 3A), suggesting increased fibroblast activation. The elevation of α-SMA was associated with significant upregulation in the mRNA expression of matrix metalloproteases (MMP)2 and 9 (*P* < 0.01, Fig. 3B), which play important roles in regulating ECM turnover. In addition, the renal protein expression of fibronectin (FN) and COL1A was also significantly increased due to CsA nephrotoxicity (*P* < 0.01, Fig. 3C).

Compared to animals with CsA nephropathy that were treated with the vehicle control, those treated with pan-LOX and LOXL2 inhibitors showed significant reductions in the protein expression of α-SMA (*P* < 0.05, Fig. 3A), suggesting reduced myofibroblast activation. The mRNA expression of MMP2 and MMP9 was also reduced in all treatment groups (*P* < 0.001, Fig. 3B). Fibrotic marker FN was downregulated by PXS-5382 and the sequential treatment (*P* for both < 0.01); while COL1A was suppressed by PXS-5505 as well (*P* < 0.05, Fig. 3C).

### Pan-LOX and LOXL2 inhibitors suppressed the renal expression of pro-inflammatory and pro-fibrotic cytokines

**Figure 4:**
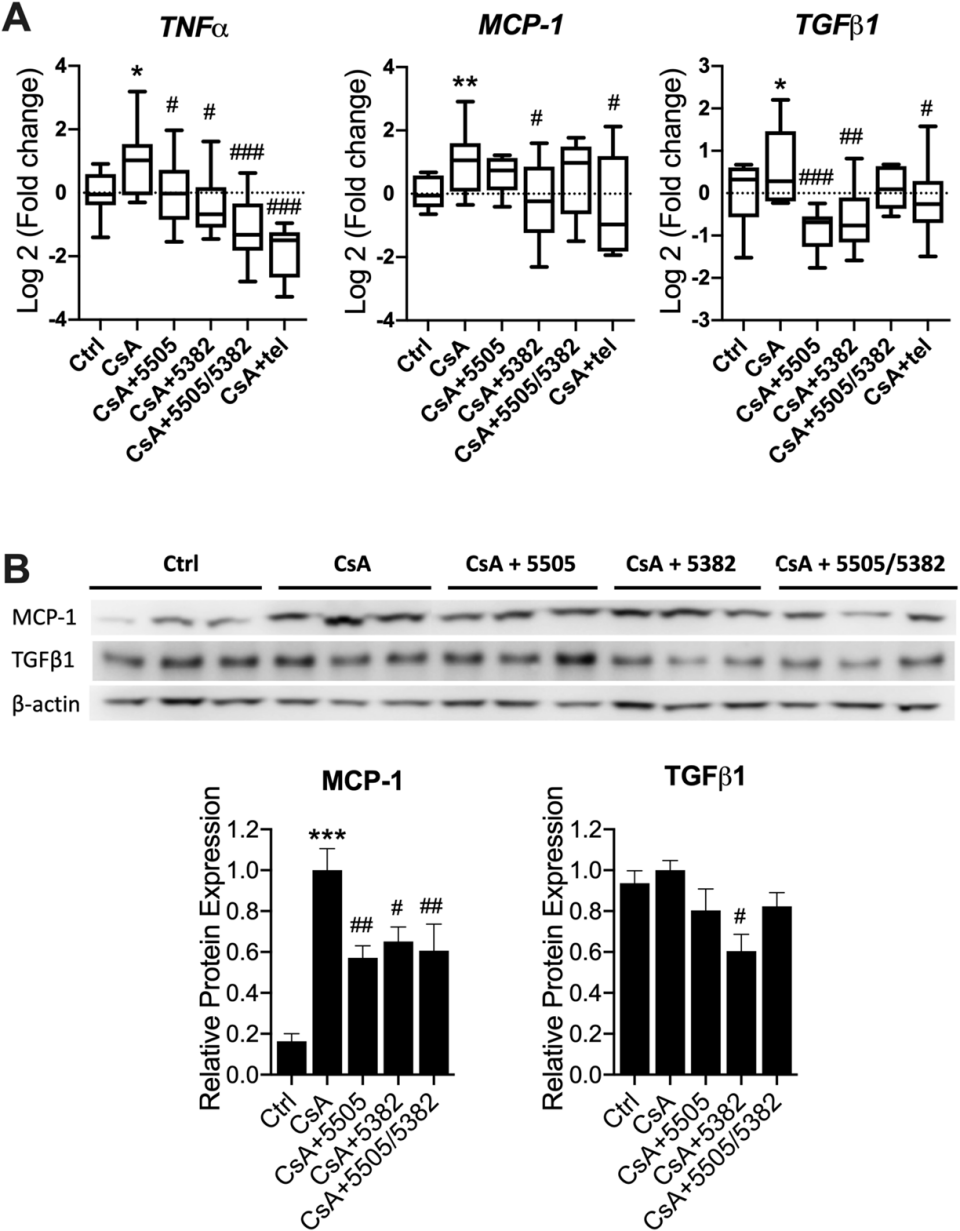
Pan-LOX and LOXL2 inhibitors suppressed CsA-induced renal inflammation and TGF-β-Smad3 signalling pathway. (**A**) mRNA expression of inflammatory markers (n = 7–10). (**B**) Representative immunoblots and protein expression of inflammatory markers (n = 6). Data are expressed as box plots with whiskers ranging from min to max for A and as Mean ± SEM for B and C. Vs Ctrl: *P < 0.05, **P < 0.01, ***P < 0.001; Vs CsA: #*P* < 0.05, ##*P* < 0.01, ###*P* < 0.001.

Renal inflammation was confirmed by qRT-PCR, which showed that the mRNA expression of TNFα and MCP-1 was significantly upregulated by CsA (*P* < 0.05 and *P* < 0.01 respectively, Fig. 4A). TGFβ1 mRNA expression was also significantly increased (*P* < 0.05), indicating a profibrotic environment. Consistent with PCR results, MCP-1 protein expression was significantly elevated in the CsA group compared to the control (*P* < 0.001, Fig. 4B). The mRNA expression of TNFα was significantly reduced by all treatments (*P* < 0.05, Fig. 4A), while that of MCP-1 was only significantly modulated by PXS-5382 and Telmisartan. TGFβ1 mRNA expression was significantly suppressed by all treatments (*P* < 0.05) with the exception of the sequential therapy. Similarly, TGF-β1 protein expression was significantly suppressed by PXS-5382 but not the other LOX inhibition regimens (*P* < 0.05, Fig. 4B).

### Pan-LOX and LOXL2 inhibitors modulate TGFβ downstream signalling pathways in a selective manner

**Figure 5:**
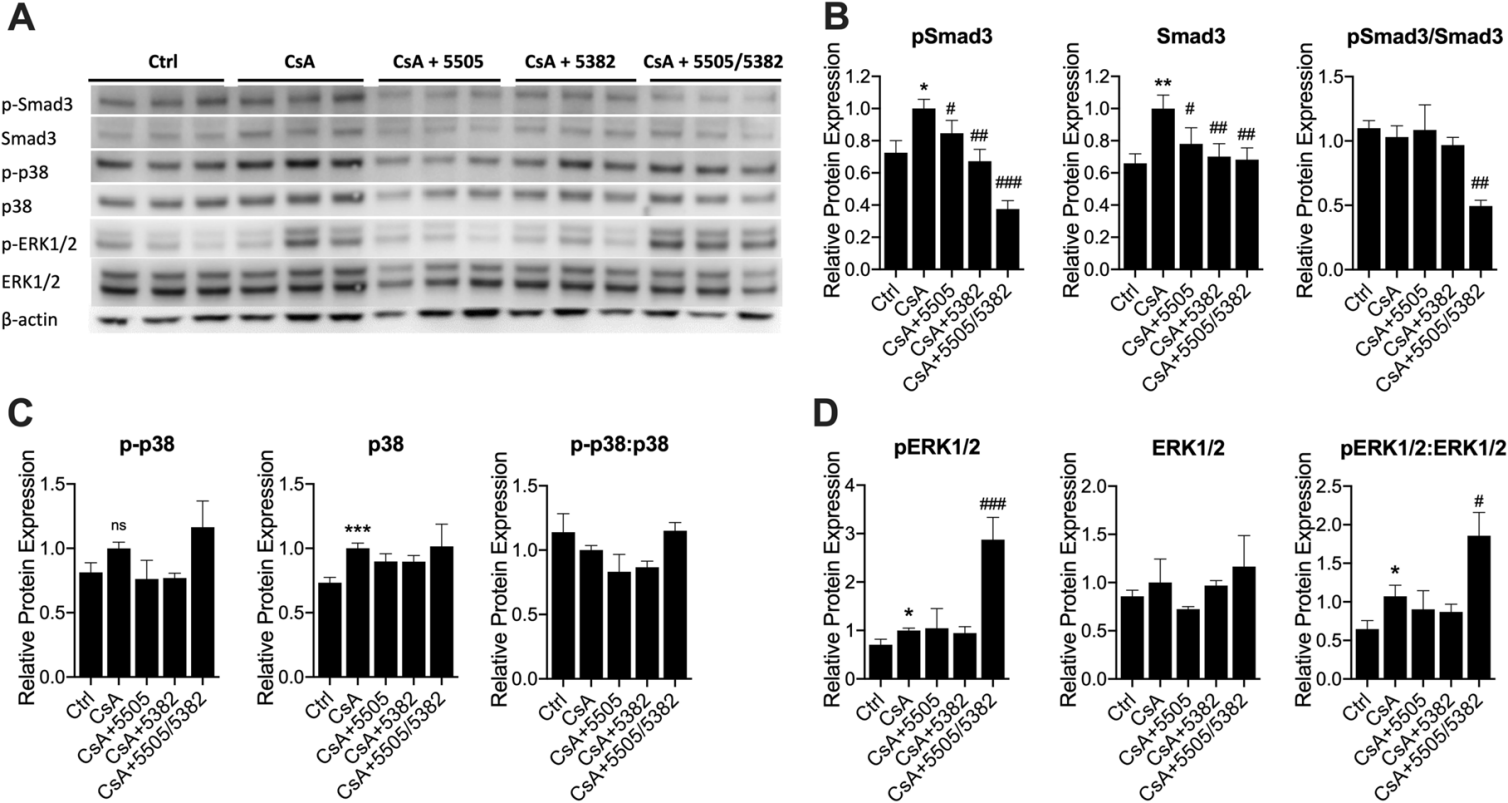
Pan-LOX and LOXL2 inhibitors suppressed Smad3 signalling pathway. (**A**) Representative immunoblots; (**B**) Protein expression of total and phosphorylated Smad3, (**C**) Protein expression of total and phosphorylated p38 MAPK; (**D**) Protein expression of total and phosphorylated ERK1/2. Data are expressed as Mean ± SEM. Vs Ctrl: *P < 0.05, **P < 0.01, ***P < 0.001; Vs CsA: #*P* < 0.05, ##*P* < 0.01, ###*P* < 0.001. (n = 6).

Although TGFβ1 protein expression was not changed by CsA administration, the expression and phosphorylation of its downstream marker Smad3 was significantly upregulated (*P* < 0.05 and *P* < 0.01 respectively, Fig. 5A,B). Apart from the Smad3 pathway, TGFβ is also known to regulate the mitogen-activated protein kinase (MAPK) pathway via a range of mediators such as extracellular signal-regulated kinases (ERK), c-Jun N-terminal kinases (JNK) and p38 MAPK^30^. In our study, total MAPK p38 expression was increased in CsA-treated mice compared to the control (*P* < 0.001, Fig. 5C). However, its phosphorylation/activation level was unchanged. In comparison, the expression of total ERK1/2 was unchanged, but its activity (p-ERK1/2) and activation levels (p-ERK1/2:ERK1/2 ratio) were significantly increased in CsA treated animals (*P* < 0.05, Fig. 5D).

All three regimens significantly suppressed the total expression and total activity (phosphorylated isoform) of Smad3 (*P* < 0.05, Fig. 5B). The sequential treatment also decreased the level of activation (reflected by the ratio of pSmad3/total Smad3) (*P* < 0.01). None of the experimental treatments had significant effects on the total activity and activation of p38 MAPK, despite a trend of reduction in animals treated with PXS-5505 and PXS-5382 (Fig. 5C). The renal levels of p38 expression and activation in the sequential treatment group were similar to the CsA group and slightly higher than in animals treated with monotherapies. Interestingly, the sequential therapy further stimulated ERK1/2 activity and activation in the kidney of CsA-treated animals (*P* < 0.05 and *P* < 0.001 respectively, Fig. 5D), while having no effects on total protein.

### The effects of Pan-LOX and LOXL2 inhibitors on CsA-induced oxidative stress

**Figure 6:**
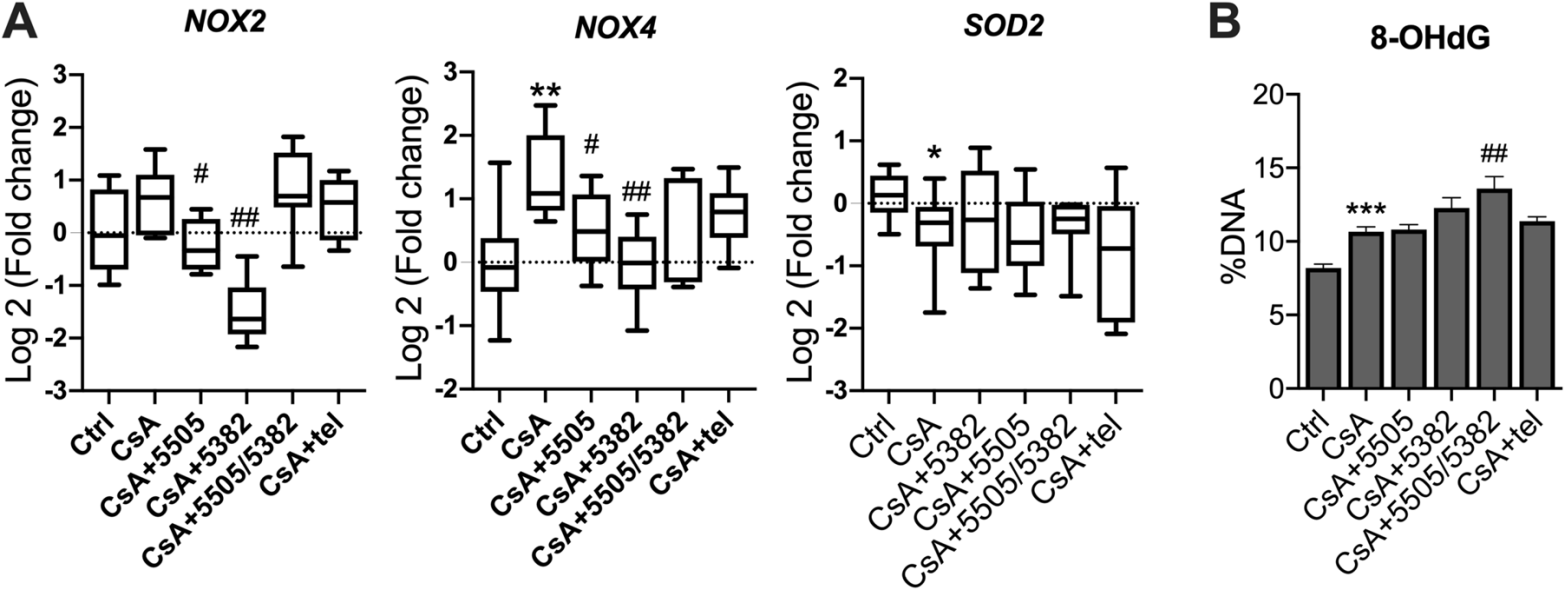
Regulation of oxidative stress markers in CsA-treated mice. (**A**) mRNA expression of oxidative stress markers (n = 7–10). (**B**) DNA oxidative damage measured by 8-OHdG (n = 6). Vs Ctrl: *P < 0.05, **P < 0.01, ***P < 0.001; vs CsA: #*P* < 0.05, ##*P* < 0.01.

Oxidative stress has been suggested to be a significant contributing factor in calcineurin inhibitor-induced nephropathy^31,32^. In this study, in comparison to the vehicle control, we found that CsA administration significantly upregulated NAPDH oxidase (NOX)4 mRNA expression (*P* < 0.01, Fig. 6A), a major source of reactive oxygen species (ROS)^33^. CsA also significantly downregulated *SOD2* mRNA expression (*P* < 0.05), a mitochondria-specific antioxidant enzyme. An imbalance between ROS and antioxidant capacity is the direct cause of oxidative damage. Indeed, the level of 8-OHdG DNA expression was significantly higher in the kidney of CsA-treated animals (*P* < 0.001, Fig. 6B). Although PXS-5505 and PXS-5382 significantly suppressed the mRNA expression of *NOX2* and/or *NOX4* (*P* < 0.05, Fig. 6A), they had no effect on *SOD2* and 8-OHdG levels. In contrast, the sequential treatment did not reduce *NOX2* and *NOX4* expression and showed a significant increase in the renal levels of 8-OHdG (*P* < 0.01, Fig. 6B).

## Discussion

Calcineurin inhibitors such as cyclosporin A (CsA) are first-line anti-rejection therapy in patients undergoing kidney transplantation. Nevertheless, CsA therapy is also a leading cause of chronic nephropathy and kidney failure. In the present study, we demonstrated that CsA- induced nephropathy is associated with LOX and LOXL2 overexpression in the kidney. We also demonstrated that LOX/LOXL2 inhibition through different regimens including 8 weeks Pan-LOX inhibitor (PXS-5505), 8 weeks of LOXL2 inhibitor (PXS-5382), or 4 weeks PXS-5505 followed by 4 weeks of PXS-5382 is beneficial in suppressing pro-fibrotic and pro-inflammatory markers in the kidney, leading to improved histology. Only the Pan-LOX inhibitor significantly improved blood urea nitrogen clearance.

Although the underlying mechanism of CsA induced nephrotoxicity is still unclear, the prevailing view is that it promotes afferent arteriole vasoconstriction and thus renal hypoxia. The bulk of evidence implicates an important role of the intrarenal RAAS in kidney functional decline and fibrotic remodelling^34,35^. In animals, renal toxicity is correlated with the level of Angiotension (Ang) II in rats treated with CsA^36^. Treatments with Ang II receptor blockers and/or ACE inhibitors have been shown to improve kidney injury although the kidney function remained unchanged^37^, suggesting that the benefit of RAAS blockade in CsA nephropathy is more relevant to long term reduction in renal fibrosis and less relevant to short-term improvement in renal function. A similar finding was seen in humans with renal transplant^38^, which aligns with the current clinical guidelines of not promoting early use of angiotensin receptor blockers to reduce longer term fibrosis as they may increase serum creatinine via haemodynamic mechanisms and can complicate recognition of early rejection. Indeed, in the present study, the Ang II receptor antagonist, telmisartan, substantially reduced kidney inflammation and fibrosis yet had no benefit and in fact slightly exacerbated BUN levels. This is consistent with a previous study in mouse^39^. On the other hand, it is not uncommon for patients with significant renal fibrosis to present with normal kidney function^40^. This emphasizes the necessity of developing novel therapeutics to simultaneously target both kidney function and fibrosis.

Regarding the effects of LOX/LOXL2 inhibition, although we are unsure why renal function was only improved in animals treated with the Pan-LOX inhibitor and not in those treated with other regimens that demonstrated higher therapeutic efficacy in improving renal structure, we believe that this could be attributed to changes in glomerular filtration. According to our data, UACR was the highest in animals treated with Pan-LOX inhibitor, which likely reflect glomerular hyperfiltration and facilitated clearance of urea nitrogen in this treatment cohort. As glomerulosclerosis remained elevated in that group, it is likely that such improvement in filtration is driven by haemodynamic mechanisms. Supporting this hypothesis, a number of studies suggested important roles of LOXs in cardiac function and vascular stiffness^41,42^, which could influence blood flow to the kidney. A limitation of this study is that renal haemodynamic parameters have not been measured to further elucidate this mechanism.

Myofibroblasts are considered as the major source of renal ECM proteins and the expression of myofibroblast marker α-SMA has been strongly correlated with renal interstitial fibrosis and functional decline^43,44^. In our study, α-SMA expression was upregulated by CsA and downregulated by LOX/LOXL2 inhibitors. Such changes are respectively associated with increases and decreases in expression of ECM markers including FN and COLs, suggesting that LOXs inhibition not only can lead to degradation of existing ECM but also deactivate myofibroblasts, thus preventing further ECM deposition to the interstitial space. Interestingly but not surprisingly, the sequential therapy appears to be superior to the monotherapies in suppressing myofibroblast activation and ECM markers. Because PXS-5505 inhibits all LOX isoforms, it was expected to be more effective in dissolving ECM protein crosslinks. However, ECM proteins play important roles in maintaining tissue structure and function, an excessive inhibition of which could be detrimental. In addition, prolonged inhibition of all LOXs isoforms could render drug resistance and trigger alternative EMT pathways. In contrast, PXS-5382 as a selective inhibitor of LOXL2 is expected to be more tolerant, thus is the more appropriate option for ECM maintenance in later stages.

In contrast to LOXs, MMP2 and MMP9 are primarily known for their catalytic function in degradation of ECM proteins. The implication of MMP2 and MMP9 in renal fibrosis is controversial as their expression/activity was reported to be both up- and downregulated depending on the stage of CKD. These MMPs may protect the kidney by promoting ECM degradation in the advanced stage; however, increases of these proteins’ expression in the early stage are pathogenic to the kidney as they impair renal structure and facilitates EMT^29,45^. This is because MMP2 and MMP9 catalyse the degradation of type IV collagen, the major component of the glomerular basement membrane. Indeed, the expression of MMP2 and 9 has been positively correlated with renal fibrosis in recent studies^46,47^. Perhaps the most relevant finding to this study was reported in kidney biopsies from transplant patients treated with CsA, which supports a positive correlation of these two markers with nephrotoxicity^48^. It has also been demonstrated that inhibition of MMP2 and MMP9 could protect transplant kidney from preservation injury^49^.

Myofibroblast activation is primarily driven by pro-fibrotic cytokines such as TGF-β that are secreted by macrophages in response to inflammation and oxidative stress. Upregulation of TGFβ has been recognised as a key driving factor of calcineurin inhibitor-induced kidney fibrosis. Mice lacking calcineurin subunit α showed increased TGFβ1 expression and nephrotoxicity^50^. In a rat model of CsA-induced nephrotoxicity, TGFβ1 expression and activity were both upregulated^51^. Apart from TGFβ, MCP-1 and TNFα are also important cytokines that facilitate inflammation and fibrosis, both of which showed increased expression in the kidney of CsA-treated mice. Several studies in other models of kidney disease have shown the crosstalk of these cytokines in orchestrating downstream inflammatory and fibrotic responses^30,52^. TGFβ, TNFα and MCP1 have all been shown to stimulate the expression and/or activation of MMP2 and MMP9^53^, which may underline the highly significant effects of LOX inhibitors on MMPs.

TGFβ1 has also been shown to mediate upregulation of LOX in fibrotic heart and kidney^54,55^. Conversely, LOX has been found to bind to TGFβ and downregulate its signalling via suppression of Smad3 phosphorylation^56^. Despite some differences in the patterns of changes between mRNA and protein expression of TGFβ, our data overall demonstrated upregulation of this pro-fibrotic marker in the kidney of CsA-treated animals, not only regarding mRNA/protein expression but also activation. Such increases were significantly attenuated by treatments with pan-LOX/LOXL2 inhibitors. Specifically, both LOX and LOXL2 inhibitors as well as the sequential therapy induced significant inhibitory effects against TGFβ1 and its downstream signalling Smad. In the sequential therapy group where the inhibition on Smad3 pathway was the most significant, the non-Smad pathways including p38 MAPK and ERK1/2 were upregulated, suggesting compensatory responses. The implication of ERK1/2 in renal disease is controversial with reports showing both protective and detrimental effects. ERK1/2 activation can mediate the effect of TGFβ on EMT stimulation^57,58^, but at the same time is also linked to anti-apoptotic effects and improved survival in response to a wide range of stimuli^59^.

Oxidative stress is also a common pathway of progressive kidney diseases. Multiple studies have shown that CsA induced ROS production and/or reduced the levels of antioxidant enzymes such as SOD2 in the kidney^31,32^. Consistent with these findings, our results demonstrated that the expression of NOX4 is upregulated and that of the antioxidant enzyme SOD2 is downregulated in the kidney of CsA-treated animals. These changes in mRNA expression are coupled with an elevation of 8-OHdG, a marker of DNA oxidative damage. Monotherapy of LOX and LOXL2 inhibitors significantly suppressed the expression of *NOX2* and *NOX4* yet had no effect on SOD2 and 8-OHdG levels. In comparison, the sequential therapy did not alter the expression of NOXs while slightly increasing 8-OHdG levels, suggesting that the sequential therapy has a distinct regulatory impact on oxidative stress markers compared to the monotherapies. Such pattern of changes in NOX expression and 8-OHdG strongly correlates with TGFβ expression and ERK1/2 activation, suggesting a potential link between TGFβ-ERK1/2 signalling and the regulation of renal oxidative stress in this model. Indeed, ERK1/2 is highly responsive to ROS accumulation and has been shown to be specifically upregulated by NOXs^60^. Considering the superiority of the sequential regimen compared to each monotherapy in preserving glomerular and tubular structures, such enhanced activation of ERK1/2 was more likely to be beneficial than harmful. It is possible that by avoiding using the same inhibitor for an extended period, which completely inhibits TGFβ and all of its downstream pathways, the sequential regimen only specifically inhibited Smad, the primary stimulator of EMT, while maintaining the other cascades that may benefit kidney cell survival and injury recovery, such as ERK1/2. Further investigations on cell death pathways could help clarify this hypothesis.

In conclusion, our study confirms that chronic CsA administration induces significant damage and functional impairment to the kidney, which can be attenuated by LOX or LOXL2 inhibitors. Collectively, LOXL2 inhibitor and the sequential therapy were more effective in treating CsA-induced renal structural changes, while pan-LOX inhibitor was more effective in improving kidney function. Although further investigations with regard to haemodynamic regulation and cell death pathways are warranted to fully understand the mechanisms of these therapeutic effects, the current study strongly suggests that LOX and LOXL2 are promising therapeutic targets for CsA-induced nephropathy in transplant patients.

## Supplementary Information

Supplementary Information 1.

Supplementary Information 2.

## Abbreviations

8-OHdG: 8-Hydroxydeoxyguanosine
α-SMA: Alpha smooth muscle actin
BUN: Blood level of urea nitrogen
COL: Collagen
CsA: Cyclosporin A
ECM: Extracellular matrix
EMT: Epithelial–mesenchymal transition
ERK: Extracellular signal‑regulated protein kinase
FN: Fibronectin
KIM-1: Kidney injury marker
LOX: Lysyl oxidases
LOXL2: LOX-like 2
MAPK: Mitogen-activated protein kinase
MCP-1: Monocyte Chemoattractant Protein-1
MMP: Matrix metalloproteases protein
NOX: NAPDH oxidase
RAAS: Renin angiotensin aldosterone system
ROS: Reactive oxygen species
PSR: Pico-Sirius Red
Tel: Telmisartan
TNFα: Tumour necrotic factor alpha
TGF-β1: Transforming growth factor beta 1
UACR: Urinary albumin creatinine ratio
