# Supplementary material for: Lysyl oxidase inhibitors attenuate cyclosporin A-induced nephropathy in mouse
Source: Sci Rep. 2021 Jun 14;11:12437. doi: 10.1038/s41598-021-91772-5 (PMC8203624; doi:10.1038/s41598-021-91772-5)
Supplement: Supplementary file 1 — Supplementary Information 1. [file 41598_2021_91772_MOESM1_ESM.docx]

**Lysyl oxidase inhibitors attenuate cyclosporin A-induced nephropathy in mouse**

Long T. Nguyen, Sonia Saad, Ying Shi, Rosy Wang, Angela S. Y. Chou, Anthony Gill, Yimin Yao, Wolfgang Jarolimek, and Carol A. Pollock

**Supplementary info**

**Table S1. Primer sequences**

| No. | Gene | Forward primer sequence | Reverse primer sequence |
| --- | --- | --- | --- |
| 1 | β-actin | CTAAGGCCAACCGTGAAAAG | ACCAGAGGCATACAGGGACA |
| 2 | Lox | CACCGTATTAGAAAGAAGCC | GTCCTTCTACTTAAGCTAATC |
| 3 | Loxl2 | AGACTGCAAATTCAACACAG | CATTTCTCTCTGTTAGCACC |
| 4 | Mcp1 | GCCTGCTGTTCACAGTTGC | CAGGTGAGTGGGGCGTTA |
| 5 | Mmp2 | GAGATCTTCTTCTTCAAGGAC | AATAGACCCAGTACTCATTCC |
| 6 | Mmp9 | CTTCCAGTACCAAGACAAAG | ACCTTGTTCACCTCATTTTG |
| 7 | Nox2 | CTACCTAAGATAGCAGTTGATG | TACCAGACAGACTTGAGAATG |
| 8 | Nox4 | AGTCTTAACCAGACATCATCC | CAGAAATCCAAATCCAGGTC |
| 9 | Sod2 | GGCCTACGTGAACAACCTGAA | CTGTAACATCTCCCTTGGCCA |
| 10 | Tnfa | CTGTAGCCCACGTCGTAGC | TTGAGATCCATGCCGTTG |
| 11 | Tgfb1 | TCAGACATTCGGGAAGCAGT | ACGCCAGGAATTGTTGCTAT |

**Table S2. Antibody information**

| **Target** | **Size (kDa)** | **Dilution (WB)** | **Cat#** | **Company** | **Address** |
| --- | --- | --- | --- | --- | --- |
| Β-Actin | 42 | 1:3000 | sc-1616 | Santa Cruz | TX, USA |
| FN | 250 | 1:5000 | ab45688 | Abcam | Cambridge, UK |
| COL1A (Pro-peptide) | 215 | 1:2000 | ab34710 | Abcam | Cambridge, UK |
| MCP1 | 28 | 1:2000 | ab8101 | Abcam | Cambridge, UK |
| TGF-β | 27 | 1:1000 | 3711 | Cell Signalling | MA, USA |
| p-Smad3 (Ser423/425) | 55 | 1:500 | 9513 | Cell Signalling | MA, USA |
| Smad3 | 55 | 1:1000 | 9520 | Cell Signalling | MA, USA |
| p-p38 MAPK (Thr180/Tyr182) | 38 | 1:1000 | 4511 | Cell Signalling | MA, USA |
| p38 MAPK | 38 | 1:1000 | 8690 | Cell Signalling | MA, USA |
| p-ERK1/2  (Thr202/Tyr204) | 44/42 | 1:2000 | 9101 | Cell Signalling | MA, USA |
| ERK1/2 | 44/42 | 1:2000 | 9102 | Cell Signalling | MA, USA |
