# Supplementary material for: Lysyl oxidase inhibitors attenuate cyclosporin A-induced nephropathy in mouse
Source: Sci Rep. 2021 Jun 14;11:12437. doi: 10.1038/s41598-021-91772-5 (PMC8203624; doi:10.1038/s41598-021-91772-5)

Supplementary data – Original immunoblot

Fig.3C - FN

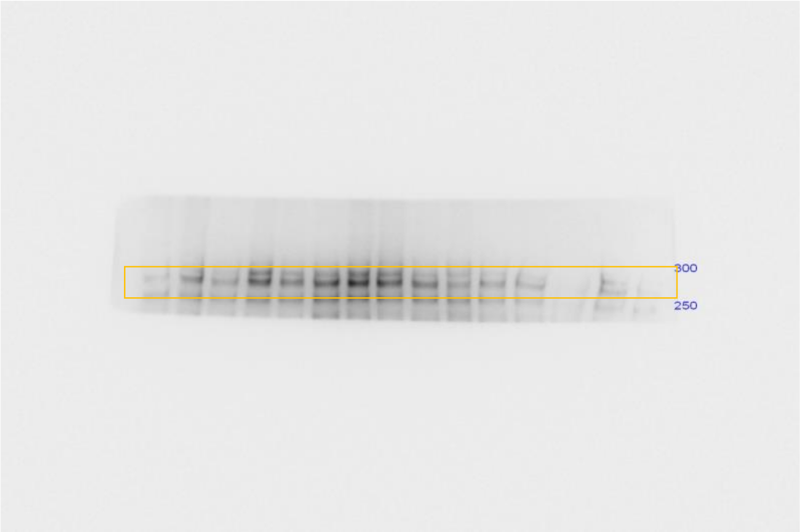

Fig.3C – β-actin (1)

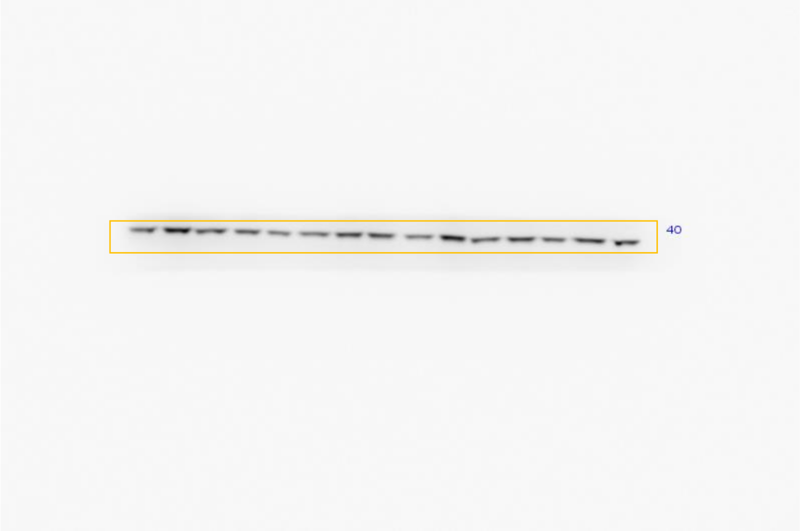

Fig.3C – COL1A

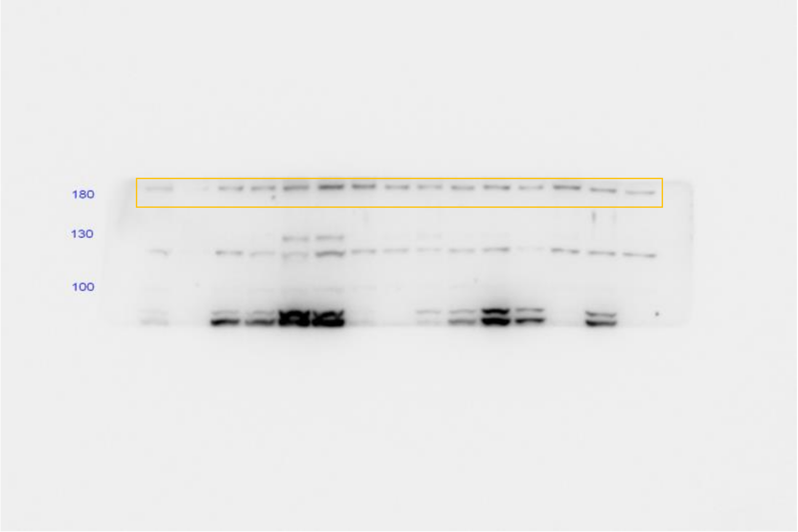

Fig.3C – β-actin (2)

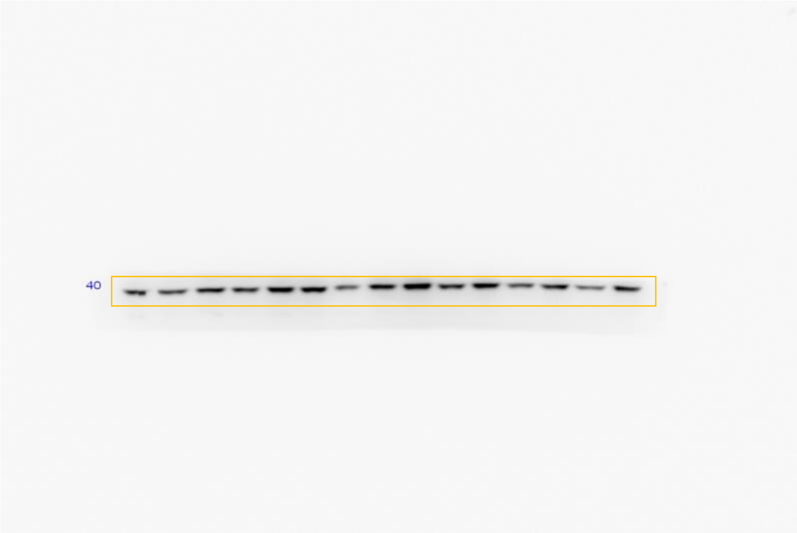

Fig.4 – MCP1

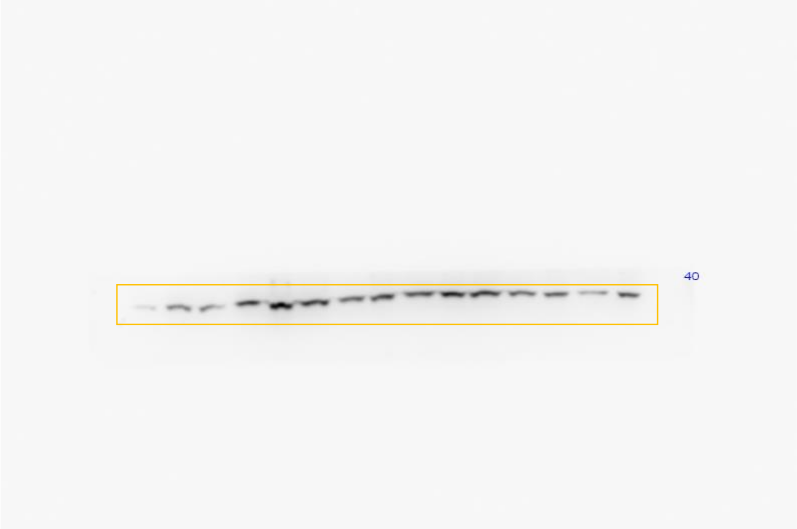

Fig.4 – TGFβ

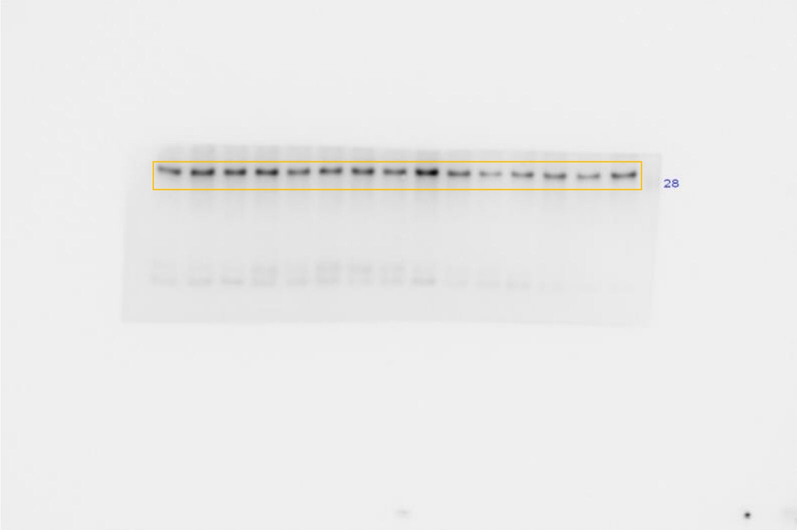

Fig.4 – β-actin

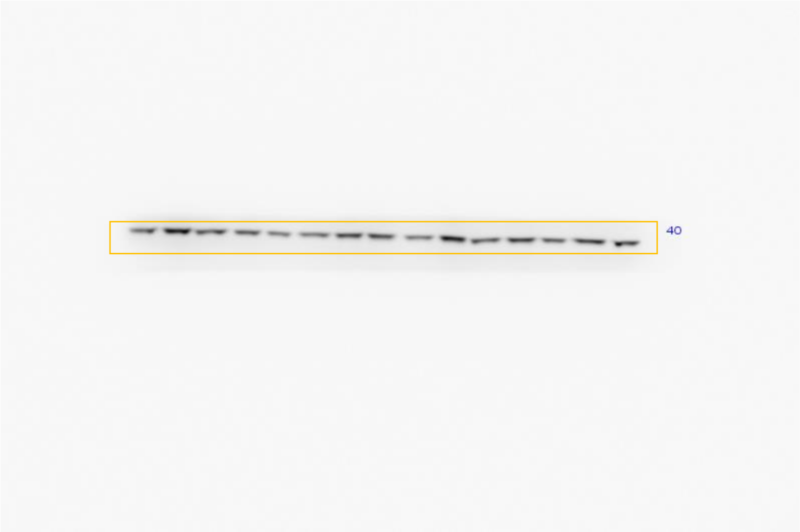

Fig. 5A – p-Smad3

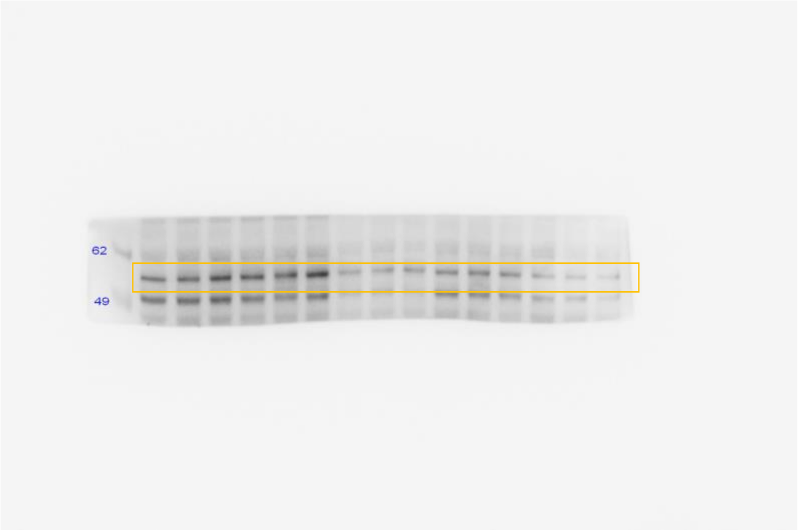

Fig.5A – Smad3

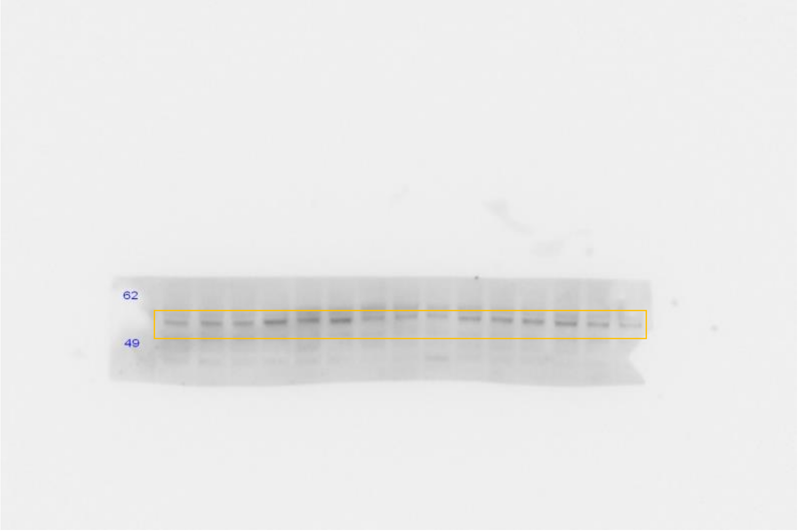

Fig.5A – p-p38

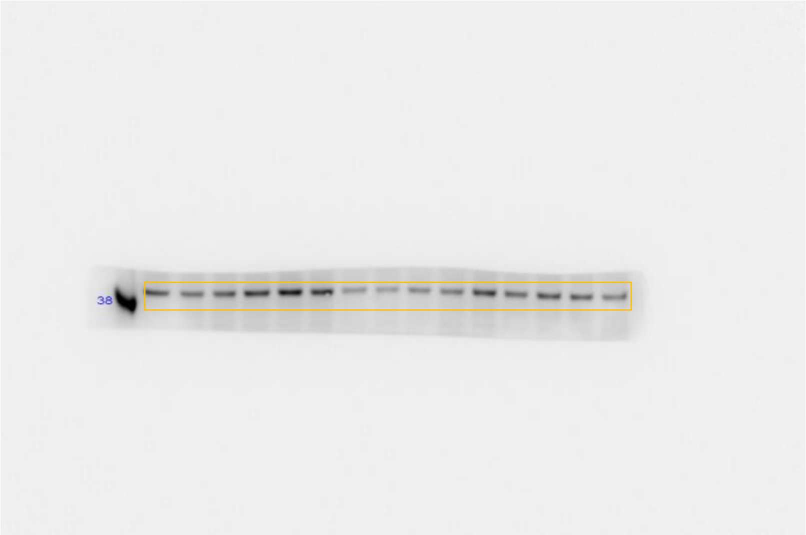

Fig.5A – p38

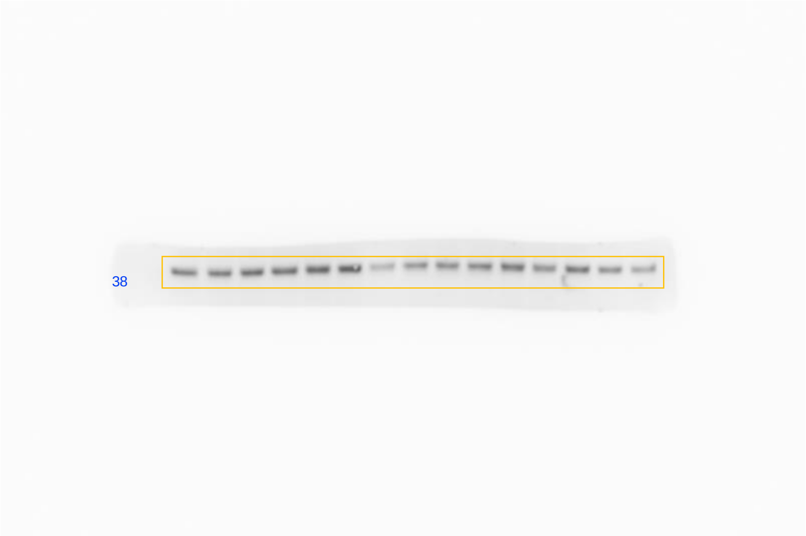

Fig.5A – p-ERK1/2

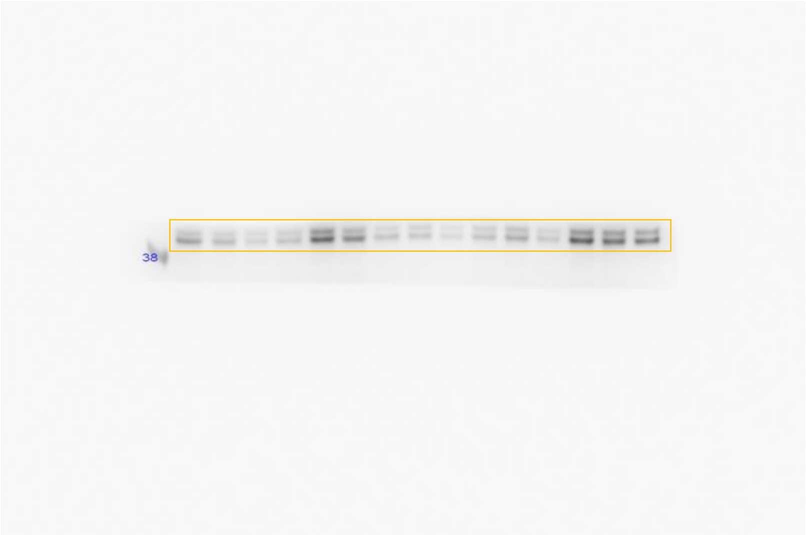

Fig.5A – ERK1/2

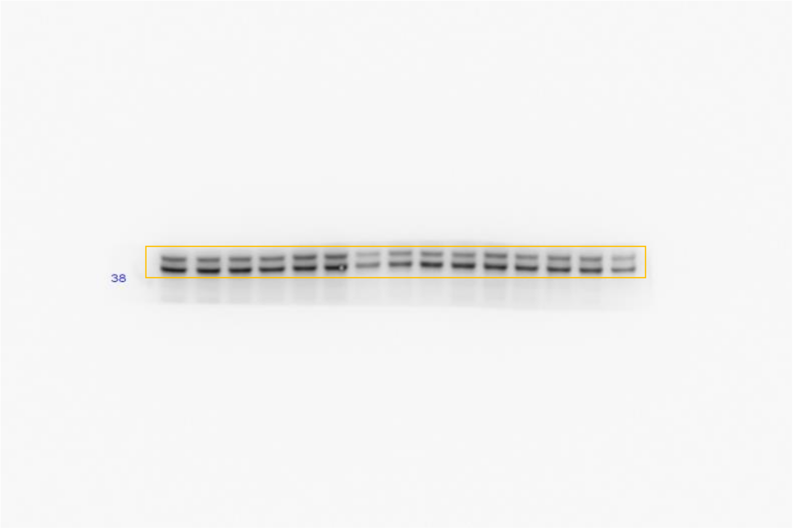

Fig.5A – β-actin

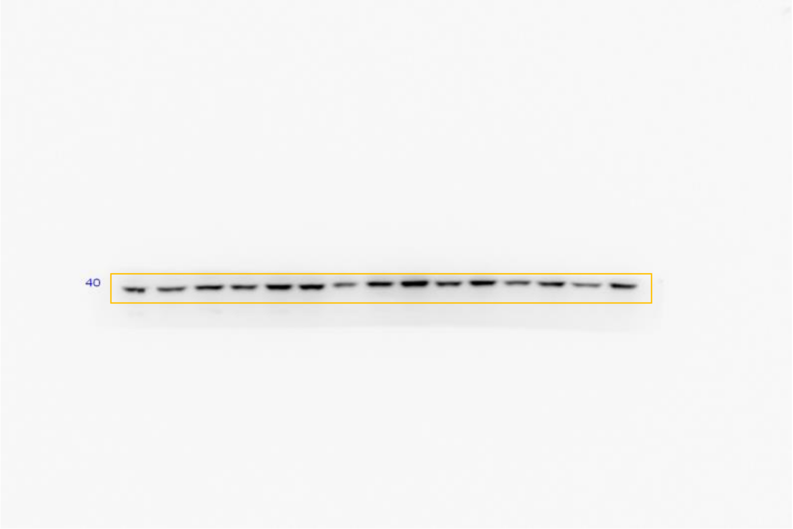

Supplement: Supplementary file 2 — Supplementary Information 2. [file 41598_2021_91772_MOESM2_ESM.pdf]
